# Generating Triangulated Macromolecular Surfaces by Euclidean Distance Transform

**DOI:** 10.1371/journal.pone.0008140

**Published:** 2009-12-02

**Authors:** Dong Xu, Yang Zhang

**Affiliations:** 1 Center for Computational Medicine and Bioinformatics, University of Michigan, Ann Arbor, Michigan, United States of America; 2 Center for Bioinformatics and Department of Molecular Bioscience, University of Kansas, Lawrence, Kansas, United States of America; Massachusetts Institute of Technology, United States of America

## Abstract

Macromolecular surfaces are fundamental representations of their three-dimensional geometric shape. Accurate calculation of protein surfaces is of critical importance in the protein structural and functional studies including ligand-protein docking and virtual screening. In contrast to analytical or parametric representation of macromolecular surfaces, triangulated mesh surfaces have been proved to be easy to describe, visualize and manipulate by computer programs. Here, we develop a new algorithm of EDTSurf for generating three major macromolecular surfaces of van der Waals surface, solvent-accessible surface and molecular surface, using the technique of fast Euclidean Distance Transform (EDT). The triangulated surfaces are constructed directly from volumetric solids by a Vertex-Connected Marching Cube algorithm that forms triangles from grid points. Compared to the analytical result, the relative error of the surface calculations by EDTSurf is <2–4% depending on the grid resolution, which is 1.5–4 times lower than the methods in the literature; and yet, the algorithm is faster and costs less computer memory than the comparative methods. The improvements in both accuracy and speed of the macromolecular surface determination should make EDTSurf a useful tool for the detailed study of protein docking and structure predictions. Both source code and the executable program of EDTSurf are freely available at http://zhang.bioinformatics.ku.edu/EDTSurf.

## Introduction

There are mainly three types of macromolecular surfaces—*van der Waals surface (VWS)*, *solvent-accessible surface (SAS)* and *molecular surface (MS)*—in molecular biology studies [1]. Because the shape and surface decide how the macromolecules interact with others, accurate determination of the macromolecular surfaces is essential for elucidating their biological roles in physiological processes. Consequently, calculations of the macromolecular surfaces from given 3D structures have found extensive uses in modern molecular biology studies, including protein folding and structure prediction [2], protein-ligand docking [3], [4], DNA-protein interactions [5], and new drug screening [6].

A variety of methods have been proposed to compute the three macromolecular surfaces. These methods can be generally categorized into two classes: analytical computation and explicit representation. For analytical computing, Connolly first presented an algorithm for calculating the smooth solvent-excluded surface of a molecule [7] (Which he called “alternative solvent-accessible surface”), where the spheres, tori and arcs were defined using analytical expressions according to the atomic coordinates, van der Waals radii and the probe radius [8]. The author also developed the Connolly's Molecular Surface Package (MSP) which was a suite of programs for computing and manipulating molecular surfaces and volumes [9]. MSMS (**M**ichel **S**anner's **M**olecular **S**urface) was later developed to compute both solvent accessible and molecular surface relying on the reduced surface [10]. There are also a number of other methods which were developed to analytically calculate the value of the exact surface area and volume [11]–[17]. Among them, Liang et al. presented a method for computing molecular area and volume based on the alpha shape theory [14] which was earlier proposed by Edelsbrunner and Muche [11]. An alpha-shape of a set of weighted points is a subset of the regular Delaunay triangulation of these weighted points. The reduced surface [10] is equivalent to an alpha-shape with an alpha value equal to zero when the radii of atoms are further inflated by the solvent radius.

Although analytical methods have the advantage of getting accurate values of surface area and volume, they are not convenient to be employed in other applications when explicit surfaces of local atoms are required for further processing. For example, local surfaces of proteins and ligands are often used for shape comparison in the docking problem. The explicit surface generation method is a grid-based approximation which uses space-filling model where each atom is modeled as a volumetric item [18], [19]. Molecules are placed onto the grids, whose width could be altered to achieve different resolution. LSMS (**L**evel **S**et method for **M**olecular **S**urface generation) used a level-set method and achieved a very fast speed [20]. Zhang et al. constructed a smooth volumetric electron density map from atomic data by using weighted Gaussian isotropic kernel function and a two-level clustering technique [21]. The authors selected a smooth implicit solvation surface approximation to the Lee-Richards molecular surface.

After the space-filling procedure, an important step is surface representation and construction. In general, macromolecular surface could be represented by parametric equations or triangular patches. Parametric representations of protein molecular surfaces are a compact way to describe a surface, and are useful for the evaluation of surface properties such as the normal vector, principal curvatures, and principal curvature directions [22]. Simplified triangular representations of molecular surfaces are useful for easy manipulation, efficient rendering and for the display of large-scale surface features. It is composed of a set of vertices and a group of triangular patches connecting these vertices. Connolly created the triangles by subdividing the curved faces of an analytical molecular surface [23]. Molecular areas and volumes may be calculated from it and packing defects in proteins may be identified. MSMS computed the triangulated molecular surfaces by sewing pre-triangulated template spheres and concave faces together.

A commonly used method to construct triangulated isosurface from 3D grid is the Marching Cube algorithm [24], which was also used in LSMS. Marching Cubes (MC) creates triangle models of constant density surfaces from 3D image data. The LSMS algorithm only considers the inside/outside attributes of each vertex and uses Marching Cubes to connect the middle point of each edge. Xiang et al. proposed an improved version of the Marching Cube method for molecular surface triangulation [25]. This new algorithm involves fewer and simpler basic building blocks and avoids the artificial gaps of the original one. Obviously, quantities like surface area and volume by grid-based algorithms may not be as accurate as that calculated by the analytical methods. However, these algorithms can generate triangular surfaces efficiently without singularities.

In this paper, we develop a new method of EDTSurf for the calculation of the three major macromolecular surfaces. We demonstrate that all the macromolecular surfaces can be universally connected with the theory of *Euclidean Distance Transform (EDT)*. Triangulated surfaces are then constructed by a variation of the Marching Cube algorithm, which forms triangles efficiently by connecting grid points directly rather than intersections of edges.

## Materials and Methods

### Macromolecular Surfaces

**Figure 1:**
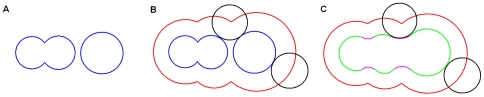
Illustration of three macromolecular surfaces in a 2D plane. (A) van der Waals surface (blue); (B) solvent-accessible surface (red); (C) molecular surface which includes contact surface (green) and reentrant surface (pink).

The definitions of the three surfaces are illustrated in Figure 1 in a 2D plane. A molecule is represented as a set of overlapping spheres, each having a van der Waals radius. The *van der Waals surface (VWS)* is the topological boundary of these spheres (see Figure 1A). The outer surface of a macromolecule binds to ligands and other macromolecules. The van der Waals surface for small molecules may describe the overall shape very well. However, since most of the van der Waals surface is buried in the interior for large molecules, it is necessary to define the other two kinds of outer surfaces as follows.

The *solvent-accessible surface (SAS)* (see the red part of Figure 1B) is defined as the area traced out by the center of a probe sphere as it is rolled over the van der Waals surface. The probe sphere is a solvent water molecule which is represented by the black circle in Figure 1B.

The *molecular surface (MS)* is a continuous sheet consisting of two parts: the *contact surface* and the *reentrant surface* [26]. The contact surface (see the green part of Figure 1C) is part of the van der Waals surface that is accessible to a probe sphere. The reentrant surface (see the pink part of Figure 1C) is the inward-facing surface of the probe when it touches two or more atoms. The molecular surface is also called the *solvent-excluded surface (SES)*, which is the boundary of the union of all possible probes which do not overlap with the molecule [10]. Molecular surface is also called the Connolly surface. It was revealed that the solvent-accessible surface was displaced outward from the molecular surface by a distance equal to the probe radius [8].

### Euclidean Distance Transform

*Distance Transform (DT)* is the transformation that converts a digital binary image to another gray scale image in which the value of each pixel in the object is the minimum distance from the background to that pixel by a predefined distance function. Three distance functions between two points 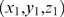 and 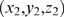 are often used in practice, which are City-block distance, Chessboard distance and Euclidean distance, i.e.
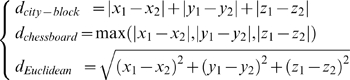
(1)


However, in our study, we found only the Euclidean distance has a direct relation to the three macromolecular surfaces (see Eqs. 7–9 below). Therefore, our discussions will be focused on this distance.

The *signed Euclidean Distance Transform (sEDT)*, which represents the displacement of a pixel from the nearest background point, is defined in Ref. [27]. The gray image after EDT, which is called *Euclidean Distance Map (EDM)*, is useful in skeleton extraction, shortest path planning and shape description. EDT can be computed efficiently by methods such as mathematical morphology [28], chain propagation [29] and boundary propagation [30]. Here, we extend the definition of Euclidean distance to the outside of an object.

Suppose the set of boundary points (or surface) of an object 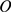 is 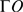. 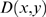 is the Euclidean distance between point 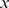 and 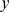. 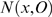 is the nearest boundary point on the surface to point 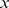. To each point 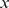, the signed Euclidean distance of 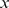 is defined as follows:
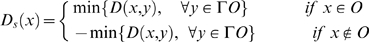
(2)


Isosurface can be extracted conveniently after the EDT. The isosurface with isovalue 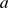 is defined as:
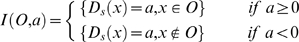
(3)


Obviously, if 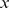 belongs to the surface, the nearest boundary point to 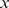 is itself and the signed Euclidean distance of 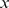 is zero. Then, we have 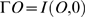.

### Macromolecular Solids

Macromolecular solids are solid bodies which are enveloped by the macromolecular surfaces. The *van der Waals solid*, *solvent-accessible solid* and *solvent-excluded solid* covered by the van der Waals surface, the solvent-accessible surface and the molecular surface are represented by 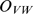, 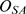 and 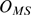 respectively. Suppose 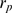 is the probe radius which is often set to 1.4 Å and there are 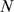 atoms except hydrogen atoms in a molecular structure. Coordinate of the *i*th atom is 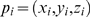 and its van der Waals radius is 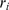. the van der Waals solid is the union of overlapping spheres and can be written as the following formula.
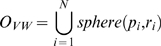
(4)where 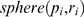 means the solid sphere with radius 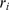 and center 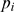. The solvent-accessible surface can also be perceived as the topological boundary of a set of spheres by increasing the van der Waals radius of each atom with the probe radius. Hence, the solvent-accessible solid can be expressed in a similar formula to that of the van der Waals solid:
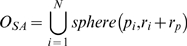
(5)


For points on the solvent-accessible surface, we define a subset 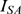 to be the intersection set in which each point can be reached by more than one solid sphere when constructing the solvent-accessible solid. That is to say, two conditions should be satisfied when defining 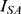: first, 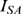 should belong to the intersection part of two or more solid spheres; second, none of the points in 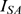 are buried inside the solvent-accessible surface.

Suppose the minimum van der Waals radius of all the *N* atoms is 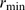. Now, we define another solid called the *minimal macromolecular solid* 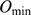. The boundary surface of the minimal macromolecular solid is called the *minimal macromolecular surface* 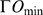.
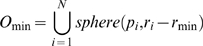
(6)


If the van der Waals radius of an atom *i* equals 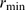, the solid sphere degenerates to a point located at 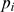. The boundary of the point 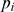 is set to be itself. If all the van der Waals radii are the same, the minimal macromolecular solid becomes a point set and the minimal macromolecular surface is the same as the minimal macromolecular solid. Otherwise, if the van der Waals radius of an atom *i* is larger than 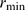, its boundary is the surface of the sphere with radius 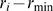.

The above three equations stand for a kind of space-filling methods which are the preliminary steps for grid-based macromolecular surface generation.

### Macromolecular Surfaces from EDT

After applying EDT to macromolecular solids as described above, the macromolecular surfaces can be treated as isosurface extracted from EDMs.
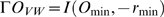
(7)

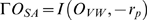
(8)

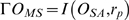
(9)


**Figure 2:**
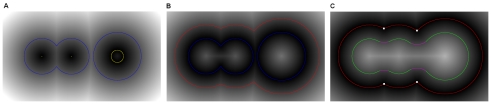
Illustration of three macromolecular surfaces from EDT in a 2D plane. (A) EDT with the minimal macromolecular surface (yellow) as the boundary. The isosurface with isovalue equaling the negative of the minimal van der Waals radius is the van der Waals surface (blue). (B) EDT with van der Waals surface (blue) as the boundary. The isosurface with isovalue equaling the negative of the probe radius is the solvent-accessible surface (red). (C) EDT with solvent-accessible surface (red) as the boundary. The isosurface with isovalue equaling the probe radius is the molecular surface which contains the surface (green) and reentrant surface (pink).

Equation (7) is elucidated in Figure 2A in the 2D plane. The gray-scale value of each pixel is the minimum distance from that pixel to the nearest boundary surface. The minimum macromolecular surface is colored in yellow and the minimum macromolecular solid is the area covered by the minimal macromolecular surface. We then apply EDT to the minimal macromolecular solid and extract the isosurface whose isovalue is 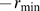. The equation means the extracted isosurface is the van der Waals surface, as shown by the blue part of Figure 2A.

Suppose the van der Waals surface (the blue part of Figure 2B) is given. We apply EDT to the van der Waals solid which is wrapped by the van der Waals surface. The isosurface with isovalue equal to 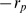 is the solvent-accessible surface, which is the red part in Figure 2B. This is the meaning of Equation (8).

Similarly, in Equation (9), we apply EDT to the solvent-accessible solid which is enveloped by the solvent-accessible surface (the red part of Figure 2C). The isosurface with isovalue equaling 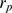 is the molecular surface, which is divided into the green and pink parts in Figure 2C. The pink part is the reentrant surface while the green part is the contact surface which overlaps with the van der Waals surface in Figure 2A. This is the first method to separate contact surface and reentrant surface.

There is another way to distinguish the contact surface from the reentrant surface without the pre-calculation of van der Waals surface, i.e.
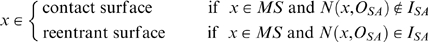
(10)


We can record the nearest boundary point 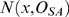 for each point 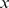 on *MS* after the EDT. If 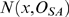 belongs to the intersection set 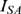, 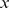 belongs to the reentrant surface; otherwise 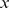 belongs to the contact surface. In Figure 2C, the four intersection points between arcs constitute the 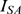, which are marked with small white blocks. The pink part belongs to the reentrant surface since the nearest boundary points are these four points.

### Algorithm Flow

**Figure 3:**
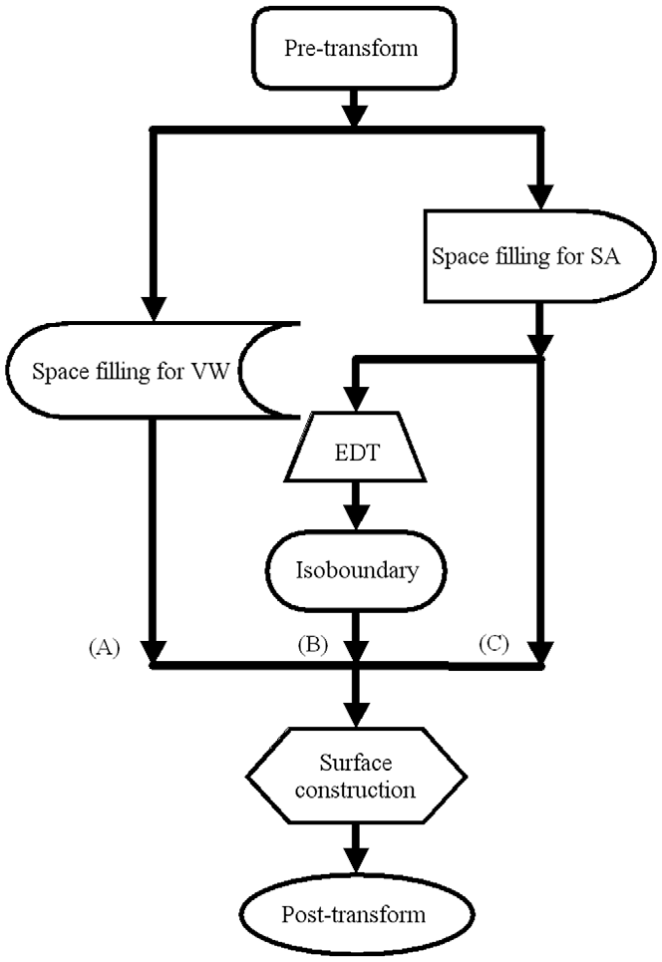
Algorithm flow chart for EDTSurf macromolecular surface construction. (A) van der Waals surface; (B) molecular surface; (C) solvent-accessible surface.

In Figure 3, we present the flowchart of the algorithm for computing the three macromolecular surfaces from 3D volumetric solids, which mainly contains five steps. The atoms of a molecular structure need to be scaled first and accommodated in a bounding box whose length is assumed to be 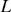. Only the grid points with integer coordinates, which are called voxels, are processed within the bounding box. The resolution is therefore 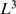. The scaled volumetric representations of 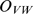, 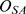 and 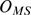 are called 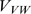, 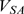
and 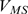 separately.

#### Step I

Translate and scale the coordinates of all the atoms in the molecular structure in order to fit them in the bounding box. After scaling, the van der Waals radius 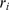 and the probe radius 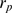 become 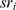 and 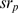.

#### Step II

To construct the van der Waals surface, treat each atom of type 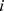 as a solid volumetric sphere whose radius is 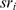. Use the space-filling method to get the scaled volumetric solid 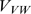. To get the solvent-accessible surface and the molecular surface, set the radius to be the summation of 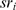 and 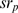. Use the space-filling method to get the scaled volumetric solid 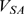.

#### Step III

To get the van der Waals surface and the solvent-accessible surface, go to step IV directly. To get the molecular surface, do EDT to the volumetric model by using Equation (9). Get rid of the voxels whose Euclidean distances are less than 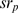. The remaining solid is 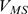.

#### Step IV

Use Vertex-Connected Marching Cube method to construct the triangulated surfaces from the volumetric models.

#### Step V

Scale and translate the generated surface back to the original size and position.

Since 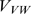 and 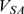 are simply unions of solid spheres, we directly use the space-filling method rather than Equations (7) and (8) to get the van der Waals surface and the solvent-accessible surface. In step II, we can speed up the space-filling process. The volumetric sphere of each type of atom can be pre-computed only once. The center of this sphere is then translated to the transformed coordinate of this atom. The voxels in the sphere are then filled. We can also record the atomic information in conjunction with the voxels.

In step III, the propagation stops when the Euclidean distance is larger than 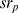. That is to say, we don't need to do EDT to the whole solid 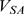. This will also accelerate the computation of molecular surface. After step III, the remaining solid is the scaled solvent-excluded solid 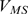, whose boundary 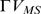 is called isobounday since it is the discretized representation of the isosurface after the EDT to 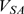.

### Triangulated Surface Construction

After we get the three kinds of macromolecular solids, triangulation is needed to construct the ultimate macromolecular surfaces. We developed the dual of the traditional Marching Cube algorithm here, which is called Vertex-Connected Marching Cubes (VCMC). The difference between them is that the vertices of the triangles in the traditional Marching Cubes are surface-edge intersections while the vertices in the VCMC are the existing grid points. When the resolution of grid is very high, there is no additional cost for real-time construction and rendering of the triangular surface by VCMC. Furthermore, the triangulation result generated by VCMC contains fewer vertices and faces than that by MC.

**Figure 4:**
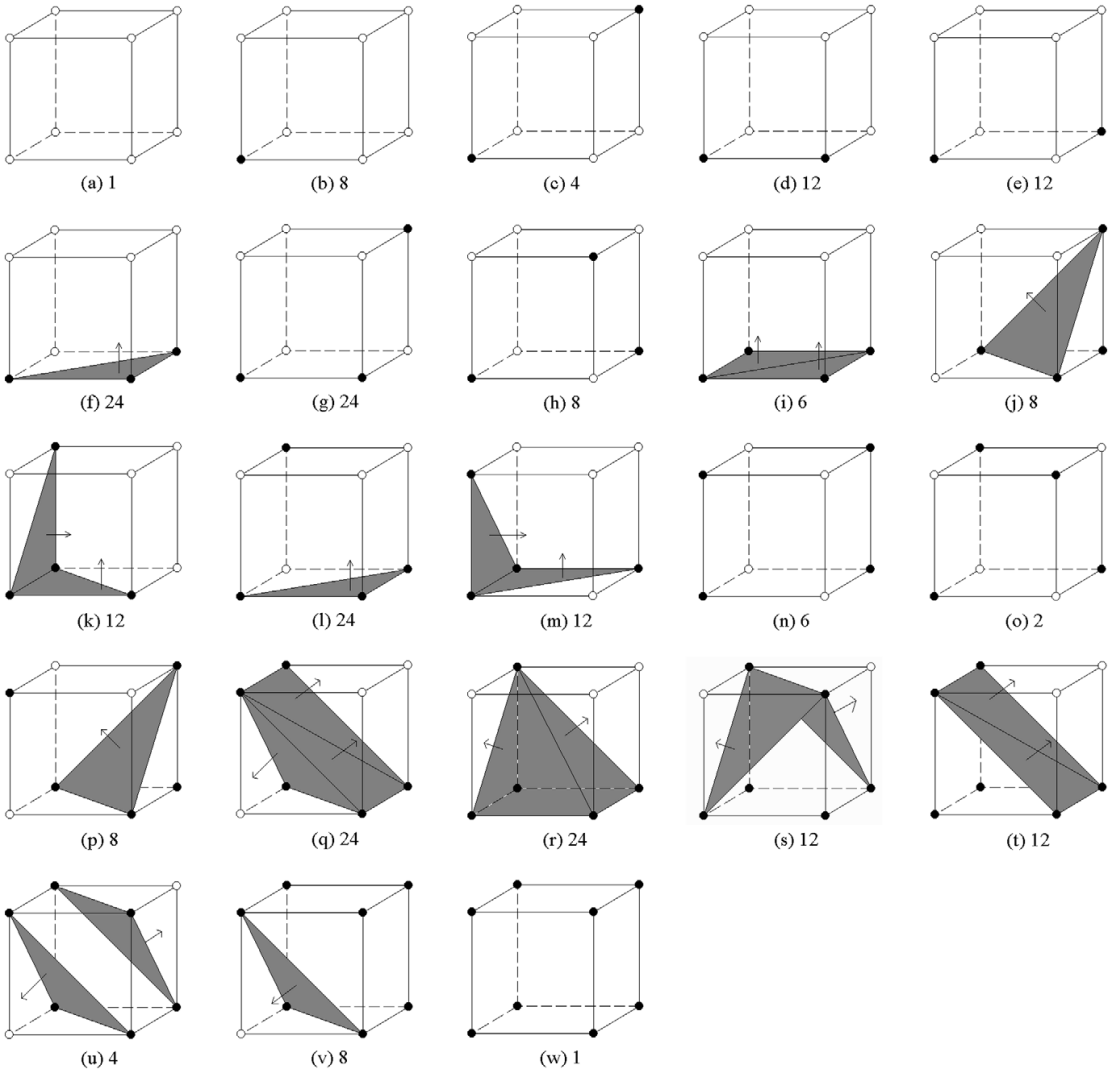
All patterns of triangulation for Vertex-Connected Marching Cubes.

For a unit cube which has eight vertices, there are totally 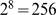 cases because each vertex may be inside or outside of the solid. We group all the cases into 23 patterns according to the symmetry of the cube in the three-dimensional space (see Figure 4). The vertex belonging to the solid is represented by a black sphere while the vertex outside the solid is represented by a white sphere. The normal vectors of the triangles are also given which point to the outside of the solid.

The number of cases for each pattern is also marked in Figure 4. Patterns (a) to (e) contain less than three vertices and cannot form any triangle. In patterns (g), (h), (n) and (o), the black spheres are separated by white spheres, so they also can't form any triangles. Pattern (w) means that all the eight vertices are inside the object. Patterns (l), (f) and (p), (j) have the similar results. All the triangles are pointing to the outside, which are shown in patterns (i), (k), (m), (q) and (u). Some black spheres in patterns (r), (s), (t) and (v) which are not involved will be considered in the neighboring cube.

All the triangles formed in Figure 4 can be further grouped into three classes based on their shapes. The edge lengths of the three triangles (two right-angled triangles and one equilateral triangle) are 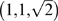, 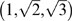 and 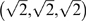 separately. Hence, the ultimate mesh surface after VCMC doesn't contain any narrow triangles, obtuse triangles and slivers. This provides a satisfactory property in numerical calculations of physical forces, such as electrostatic interactions [31].

## Results and Discussion

### Triangulated Surfaces and Computation of Area and Volume

**Figure 5:**
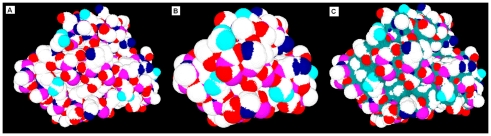
Three macromolecular surfaces of protein 1eca. (A) van der Waals surface (B) solvent-accessible surface (C) molecular surface.

Molecular structures in the RCSB Protein Data Bank (PDB) are mainly obtained by the techniques of X-ray crystallography and nuclear magnetic resonance spectroscopy. Figure 5 shows an example of the Erythrocruorin protein (PDB ID: 1eca) with surfaces generated by EDTSurf using a length of the bounding box of 256. Different colors of the surface patches represent different type of atoms. The reentrant surface is colored in teal blue in Figure 5C. The contact surface is the same as that in van der Waals surface in Figure 5A. In our algorithm, the computation of surface area and volume is straightforward, i.e. the surface area is the summation of all the triangular patches while volume is the product of the number of grid points in the macromolecular solid and the unit volume for each point.

**Figure 6:**
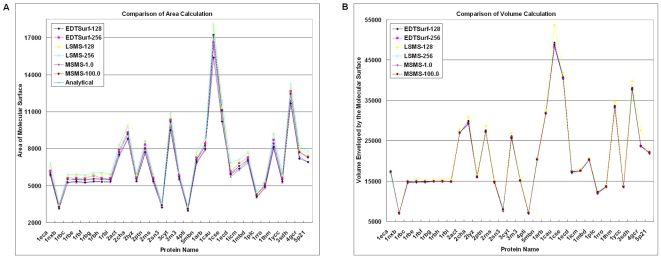
Comparison of accuracies of molecular surfaces at two different resolutions. Left panel is the numerical surface areas and analytical surface area of 31 proteins; right panel is the corresponding numerical volumes enveloped by the molecular surfaces.

Since the area of surface can be analytically calculated by MSMS [10], we try to evaluate the accuracy of EDTSurf based on the result from MSMS. For the numerical volume calculation, we set the vertex density for MSMS up to 100.0 vertex/Å^2^. The purpose of such a high vertex density is to make the numerical volume calculation of MSMS as close as possible to the exact value. When the MSMS program fails to generate output results with such a high vertex density, however, we set the vertex density to a lower value. As a control, we also run LSMS [20], another grid-based program, on the same set of protein molecules. For the convenience of comparison, we set the radii of the atoms, the probe radius (1.4 Å) and the size of bounding boxes (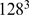 and 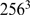) for volumetric manipulation at the same values in EDTSurf and LSMS. We also run MSMS with its default vertex density 1.0 vertex/Å^2^. In Figures 6A and 6B, we present the area and volume calculation results by EDTSurf, LSMS and MSMS for 31 test proteins that have been used in Ref. [14]. In Figure 6A, we also indicate the analytical surface area from MSMS. Compared to LSMS, the area and volume calculated by EDTSurf are better in 23 and 26 out of 31 cases. A similar tendency is seen at resolution 256^3^. The average relative errors of these algorithms are listed in Table 1.

**Table 1:** Average relative errors of area and volume of molecular surface at two different resolutions calculated by EDTSurf, LSMS [20] and MSMS [10].

Method	Resolution	Average relative error of area	Average relative error of volume
EDTSurf	128^3^	3.96%	1.18%
	256^3^	1.99%	0.48%
LSMS	128^3^	6.10%	3.57%
	256^3^	7.87%	0.84%
MSMS	1.0	4.56%	0.72%
	100.0	0.45%	-------

If the vertex density is very high, the difference between numerical and analytical surface calculations by MSMS is small, i.e. 0.45%. If we take the values of the analytical area and the high-accurate numerical volume by MSMS as the golden-standard, the relative errors for surface and volume are 3.96% and 1.18% for EDTSurf at the resolution 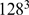, both of which are lower than that of LSMS (i.e. 6.10% and 3.57%) at the same grid resolution. The errors will become smaller when the grid resolution increases, which of course takes a longer CPU time. In Table 1, we also calculate the surface and volume at 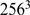, where the errors are reduced to 1.99% and 0.48%, respectively, which are still much lower than that by LSMS at the same grid resolution (7.87% and 0.84%, respectively). These data demonstrate that the representation by Euclidean Distance Transform can be more accurate than the level-set-based approach [20] at the same resolution.

### CPU Time and Memory Use

Except for the accuracy of surface, an important requirement of the surface calculation programs is the increase of speed and decrease of memory cost. The time spent on computing the molecular surface in EDTSurf is composed of three parts: generation of scaled solvent-accessible solid 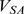, EDT to the isoboundary of 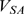, and triangulated surface generation by Vertex-Connected Marching Cube algorithm. Atom type of each voxel is not recorded in this experiment.

For testing the computer cost, we apply our algorithm to 15 large protein molecules taken from the PDB, which have 27,375 to 97,872 atoms. This set of proteins has also been used by Can et al. to compare their algorithm LSMS with three other programs, including the MSMS, which is integrated in UCSF Chimera [32]. Their result shows that LSMS algorithm is the fastest one among them. Here, we compare our algorithm with LSMS where the same parameters are exploited, i.e. the probe radius (1.4 Å), the van der Waals radii, size of bounding box (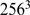). We also report the results by running MSMS whose vertex density is relatively low (1.0 vertex/Å^2^ here). Both the three algorithms run on a Microsoft Windows XP machine with Intel Pentium 4 Processor at 1.9GHZ and 768 MB of RAM.

As shown in Table 2, EDTSurf only costs on average about 12 seconds for calculating the surfaces which is about 1.6 times faster than LSMS. Also, the memory of EDTSurf is low. In this experiment, the average RAM request for these molecules is 152 MB, which is about 2.1 times less than that by LSMS. It is also comparable to the computational geometry method MSMS.

**Table 2:** CPU time and memory use for molecular surface generation by EDTSurf, LSMS [20] and MSMS [10].

Protein	#Atoms	Surface generation time (s)/maximum memory use (MB)		
		EDTSurf	LSMS	MSMS
1a8r	27375	4.25/71.33	16.28/288.36	4.10/31.22
1h2i	32802	6.60/78.94	17.20/299.91	12.83/94.99
1fka	34977	13.85/208.72	19.21/328.77	11.94/116.42
1gtp	35060	5.88/65.10	17.17/298.07	30.80/110.26
1gav	43335	13.21/244.46	18.07/309.66	18.21/132.68
1g3i	45528	11.31/121.66	19.10/319.21	46.95/145.66
1pma	45892	17.85/159.73	20.68/333.26	19.80/146.19
1gt7	46180	7.00/103.88	17.10/296.31	14.53/106.38
1fjg	51995	12.86/192.19	19.34/321.88	44.91/183.89
1aon	58884	14.36/140.13	20.71/335.77	63.59/191.70
1j0b	60948	11.84/196.99	17.96/308.77	72.83/167.54
1ffk	64281	16.62/200.09	21.00/356.07	70.01/270.90
1otz	68620	17.63/218.82	21.40/331.28	52.21/165.49
1ir2	87087	10.12/105.05	18.41/309.58	53.93/159.28
1hto	97872	15.32/172.59	20.95/333.08	35.15/250.49
avg.	53389	11.91/151.98	18.97/318.00	36.79/151.54

The speed of EDTSurf and LSMS are both dependent on the size of bounding box while that of MSMS relies on the number of atoms and vertex density. If the triangulation result contains singularities in each round, MSMS will change the radii of some atoms and perform several rounds of computations. This is partly the reason for the expensive time cost of MSMS for most of proteins in the Table 2. Moreover, if the vertex density is higher, the time cost for surface triangulation will be higher in MSMS.

Since the computational complexity of MSMS is *O(Nlog(N))* (*N* is the total number of atoms in the molecule), MSMS is not efficient for large supramolecular complexes. Both EDTSurf and LSMS have the computational complexity *O(L^3^)* (*L* is the length of the bounding box). They will be slower than MSMS when handling molecules with a small number of atoms.

### Cavity Detection

Protein cavities can be empty or water-containing. They can be within domains, between domains, or between subunits. The buried water molecules in the internal cavities contribute to protein stability. This is because the water-filled cavities are important for modulating residues surrounding the cavities. Cavities can help us to locate the proton transport pathway in the membrane protein [33].

After the triangulated surface generation, one part of the molecular surface is in contact with outside space while the other part is buried inside the molecular solid. Cavities are those formed by the inner molecular surface. Since molecular surface is propagated from solvent-accessible surface by our method, it can be seen that the number of cavities in the molecular surface obtained is equal to that in the solvent-accessible surface.

In Table 3, we compare our algorithm on 6 protein structures with LSMS and MSMS for the cavity detection. The bounding boxes for EDTSurf and LSMS are set to be the same (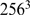). The probe radius is 1.2 Å. The vertex density for MSMS is 100.0 vertex/Å^2^. It is shown in Table 3 that EDTSurf detects fewer cavities than LSMS and MSMS. This is because some small cavities enveloped by the molecular surface may be filled when constructing the solvent-accessible solid. Using the space-filling method, the solvent-accessible solid occupies more spatial space than the van der Waals solid because each van der Waals radius is enlarged by the probe radius. These small cavities can't be formed after Euclidean Distance Transform and molecular surface generation. Clearly, cavities which can't be detected by our method are too small to accommodate any solvent water molecules. They are filtered out naturally by our method and not necessary to be considered further. The operations which first increase the radius then do EDT are equivalent to the dilation and erosion in mathematical morphology. A dilation followed by an erosion is called close operation which helps to close up breaks between van der Waals surface here.

**Table 3:** Number of cavities and the cavity volume of the molecular surface by EDTSurf, LSMS [20] and MSMS [10].

Protein	#Res	No. of cavities/cavity volume (in Å^3^)		
		EDTSurf	LSMS	MSMS
2act	218	14/533.00	16/514.66	18/573.858
2cha	248	7/347.44	19/529.91	20/587.81
2lyz	129	5/220.76	6/190.47	11/274.44
2ptn	230	7/411.29	14/608.94	20/680.45
5mbn	154	4/168.41	8/298.52	13/293.94
8tln	318	14/441.75	29/642.06	42/942.91

In Table 4, we calculate the volumes of seven cavities in the disordered domain of trypsinogen (PDB ID: 2ptn). The residues around each cavity are also tabulated, which are represented by the abbreviation of the residue names and residue sequence numbers. Cavity with small volume definitely has fewer residues than that with large volume.

**Table 4:** Residues around the seven cavities of protein 2ptn calculated by EDTSurf.

Cavity	Volume (in Å^3^)	Contributing residues
1	186.00	G23, N25, T26, V27, P28, Y29, Q30, V31, L46, L67, G69, E70, D71, R117, V118, W141, L155
2	50.25	Q30, H40, G43, S139, G140,W141, G193, D194, G197
3	24.07	Y29, L137, S139, P198
4	13.19	A160, C136, I138, A183, V199
5	39.43	S45, V53, G196, G197, P198, L209, I212
6	85.17	L99, N100, N101, D102, N179, M180, S214, W215, V227, Y228, T229
7	13.19	

**Figure 7:**
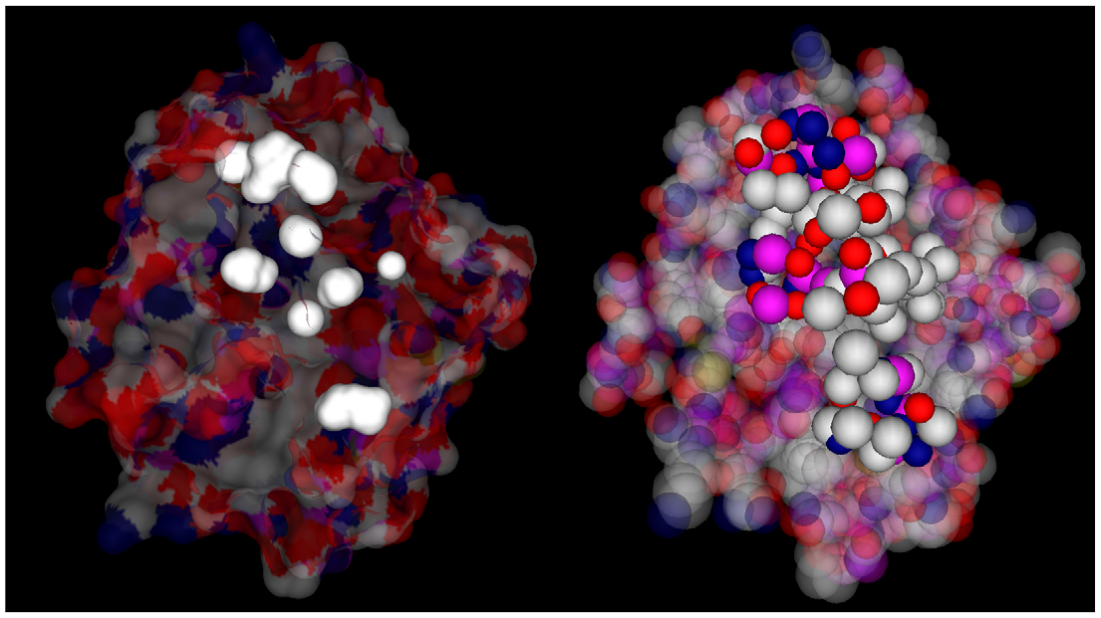
Cavity detection of protein 2ptn. Left panel is the outer molecular surface and cavities of the protein; right panel shows the atoms around the cavities.

In the left panel of Figure 7, the outer molecular surface is set to be transparent so that we can see the inner cavities clearly. We get the atoms which contribute to the outer surface and the inner surface in the right panel of Figure 7. Each atom is represented by a van der Waals sphere colored in terms of its atom type. This helps us to find the atoms which are related to the stability of cavities clearly.

### Isosurface Extraction

**Figure 8:**
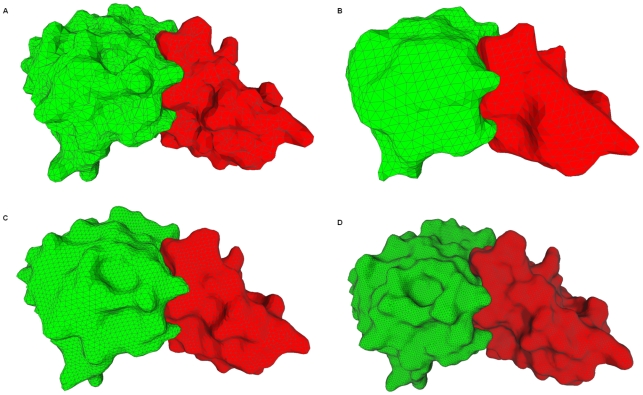
Molecular surface of a complex with the PDB ID 1brs. Chain A is in blue and chain D is in red. (A) MSMS [10] triangulation result, 9910 vertices and 19816 faces, vertex densities 1.0 vertex/Å^2^; (B) 2874 vertices and 5740 faces, resolution 32^3^; (C) 12880 vertices and 25752 faces, resolution 64^3^; (D) 55873 vertices and 111738 faces, resolution 128^3^.

Quantitative measures such as the area and the volume of molecular surface will be more precise if the grid resolution is higher. In Figures 8B, 8C and 8D, we compute the molecular surface of a large complex at three different resolutions (32^3^, 64^3^, and 128^3^) to see the visual effects. We also compare our generated surfaces with the molecular surface (see Figure 8A) by the MSMS method using its default options. At the three resolutions, the shapes are well conserved. From the figure, we can see that the surfaces are very similar to that in Ref. [10] even in a low resolution (compare Figures 8A and 8B). The two domains of the complex form the bound docking. The two complementary parts of the two domains which contact each other have very similar surface shape.

The molecular surface obtained with our approximation method approaches to the accurate analytical surface when the resolution is increased. From Figure 8, we can see that calculations with a resolution equal to 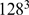 are accurate enough for most of the applications. It takes very little CPU time and memory space for computing the molecular surface at this resolution.

**Figure 9:**
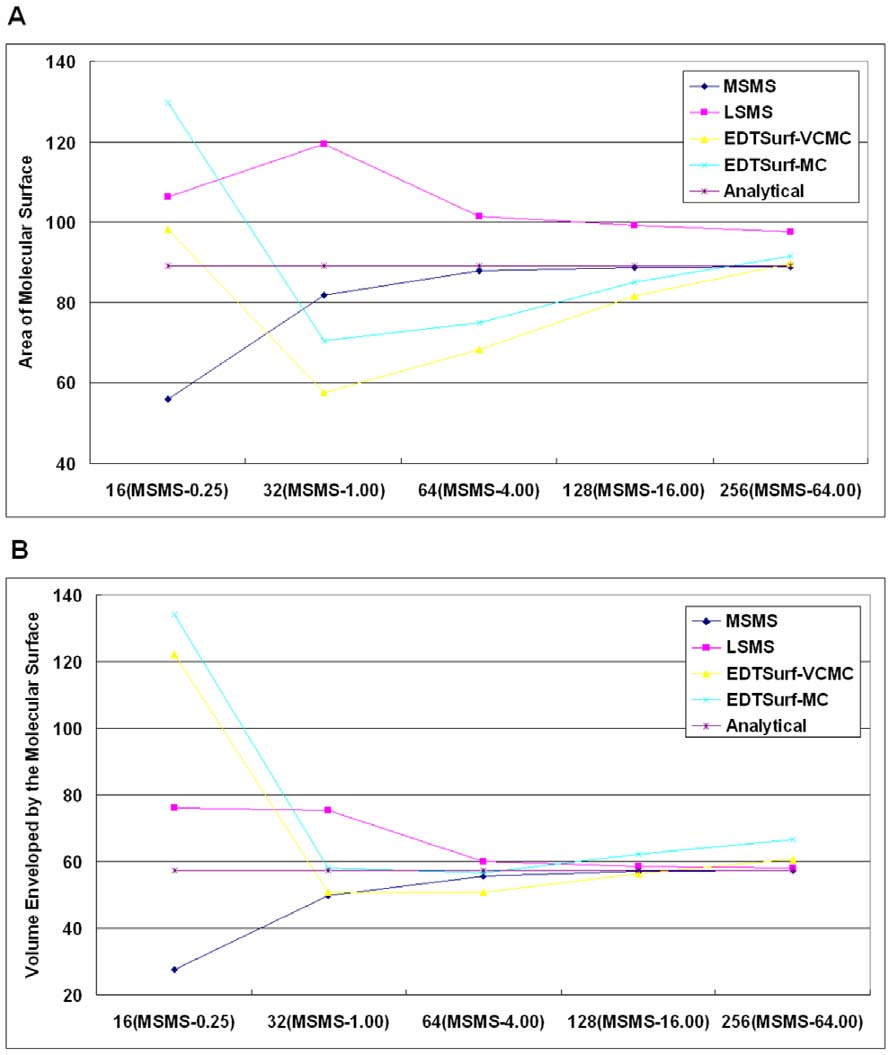
Mesurements of molecular surfaces at different resolutions. (A) area; (B) volume.

Because EDTSurf and LSMS are based on the volumetric manipulation and the surface is only an approximation to the actual analytical surface, it is interesting to examine whether and how the calculations of the gird-based methods approach to the real value of the surface and volume. Here, we use the three atoms in Figure 1 as an example to check the result of EDTSurf, LSMS, and MSMS. Numerical values of area and volume calculated by the three algorithms at five different resolutions are presented in Figures 9A and 9B. The analytical surface area and volume are 89.093 and 57.505 separately. The length of bounding box in EDTSurf and LSMS varies from 16 to 256 while the vertex density of MSMS changes from 0.25 to 64. For EDTSurf, we also compare the surfaces generated by MC and VCMC. At the lowest resolution, overall shapes of the molecular surfaces by all the four algorithms (MSMS, LSMS, EDTSurf-MC and EDTSurf-VCMC) are not kept, so they all have great difference to the analytical value. Surface by MSMS converges to the real surface more quickly than the other three methods. This is because MSMS gets the sampling vertices directly from spherical surface. Surface area and volume by MC are always larger than that by VCMC because MC connects the middle point of each edge while VCMC connects grid points which are only inside the object. However, their difference will be smaller when the grid resolution increases. Both the surface area and volume by EDTSurf and LSMS will converge to some values which are larger than the analytical value, although EDTSurf is closer to the analytical results. This is partially because the surface-area-to-volume ratio for an object with triangulated surface is larger than a smooth object.

**Figure 10:**
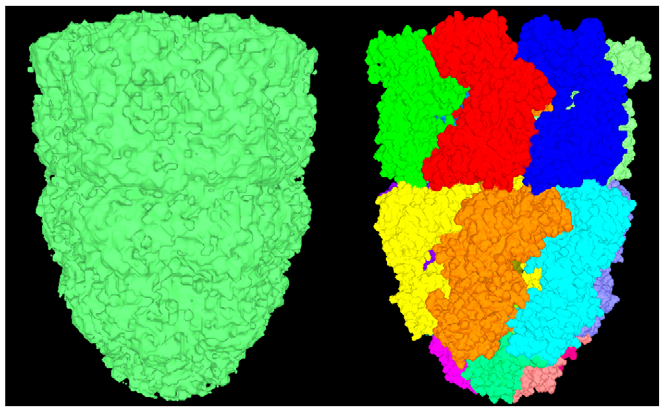
Molecular surface of a chaperonin. Left panel is the isosurface of electron microscopy volume data (EMDB ID: 1180); right panel is the molecular surface of PDB data (PDB ID: 2c7c).

There is another type of macromolecular structures which are reconstructed from electron microscopy (EM) images. On the left panel of Figure 10, we use a density map of a complex of the double-ring chaperonin GroEL and its lid-like cochaperonin GroES (EMDB ID: 1180). Its isosurface is constructed by the VCMC method after setting a threshold to the density map. The corresponding PDB file is 2c7c, which has 21 chains. The molecular surface is constructed as shown on the right panel of Figure 10, which is then segmented according to the chain information. We can see that the complex is distributed in three layers and has 7-fold symmetry. The overall shapes of the two structures are very coincidental.

### MC vs. VCMC

**Figure 11:**
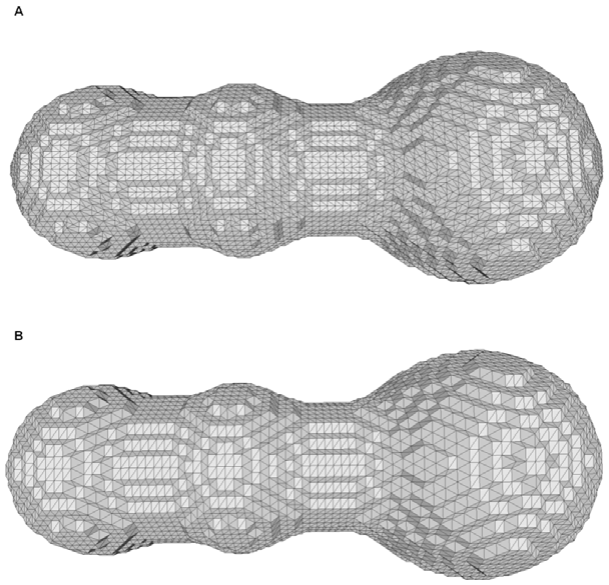
Molecular surfaces of three atoms at the resolution 128^3^. (A) generated by EDTSurf-MC; (B) generated by EDTSurf-VCMC.

In Figure 11, we show the molecular surfaces of the three atoms generated by MC and VCMC at the resolution 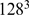. In general, their overall shapes are quite similar. However, the numbers of vertices and faces in Figure 11B are 5958 and 11912, which are only half of that (11130 and 22256) in Figure 11A. Hence, VCMC has the advantage of saving storage space when describing mesh surfaces with similar shapes.

We also compare the efficiency of MC and VCMC algorithms on the isosurface extraction for 18 EM density maps. The average CPU time by VCMC (0.54s) is about 1.4 times faster than the MC algorithm (0.75s).

As discussed in [31], a correctly triangulated mesh should be in the 2D manifold and satisfy the Euler Characteristics. Each edge is shared by exactly two triangles. The number of faces should have a general relationship with the numbers of vertices, components, genuses and voids. We checked the mesh generated by VCMC and found that only when the scaling factor is very small (surface is very rough), there would be some singularities, such as one vertex or one edge shared by two components, duplicated faces with opposite normals. MC also has such problems in many cases. Hence, we also support an additional component to check the Euler Characteristics and correct the irregular part. For example, mesh surfaces in Figures 8B, 8C and 8D are all obey the Euler Characteristics.

When we add one run of Laplacian smoothing to the generated surface, each mesh vertex is moved to the centroid of the surrounding mesh vertices which are topologically connected. This post-processing step will make the mesh surface closer to the smooth continuous surface in some degree.

### Conclusions

We have developed a new method, EDTSurf, for calculating three major macromolecular surfaces based on the method of Euclidean Distance Transform. Triangulated surfaces are then constructed by using Vertex-Connected Marching Cube method. The two parts of the molecular surface which are the contact surface and the reentrant surface can be efficiently distinguished. The resolution of the grid system can be controlled flexibly. The area and the volume of molecular surface are calculated accurately. Surfaces of the interior cavities and their surrounding atoms could be detected. Moreover, compared with the methods in literature, the EDTSurf algorithm is faster in speed and consumes less memory, especially when the number of atoms in the molecule is large.

As an application in protein structure prediction, we have applied EDTSurf to generate the solvent-accessible surface area of each residue for all proteins in the PDB library. This provides an essential frame for matching the predicted solvent accessibility with that of template structures in our fold-recognition algorithm [34]. As alternative extensions of the proposed Euclidean Distance Transform technique, distance functions other than the Euclidean distance can also be considered for the surface generation. This will result in new surface applications. For example, a solvation surface using Gaussian isotropic kernel function can approximate molecular surface [21]. It is hoped that the generated surfaces have use on different aspects of molecular biology studies.

Although the illustrations have been given for proteins molecules throughout the paper, the surface of any other macromolecules such as RNA or DNA can also be calculated using EDTSurf. The source code and executable package of EDTSurf are freely available at http://zhang.bioinformatics.ku.edu/EDTSurf. All the images have been generated by the MVP (**M**acromolecular **V**isualization and **P**rocessing) software which is also freely downloadable at http://zhang.bioinformatics.ku.edu/MVP.
